# Long-Term Use of Antiplatelet Therapy in Real-World Patients with Acute Myocardial Infarction: Insights from the PIPER Study

**DOI:** 10.1055/s-0038-1676529

**Published:** 2018-12-21

**Authors:** Luca Degli Esposti, Valentina Perrone, Chiara Veronesi, Stefano Buda, Roberta Rossini, on Behalf of a LHUs Group

**Affiliations:** 1 CliCon S.r.l., Health, Economics, and Outcomes Research, Ravenna, Italy; 2 USC Cardiologia, Ospedale Santa Croce e Carle, Cuneo, Italy

**Keywords:** acute myocardial infarction, antiplatelet therapy, adherence to treatment

## Abstract

The aim of this study was to assess long-term drug adherence and prognosis in real-world patients discharged on dual-antiplatelet therapy (DAPT) after acute myocardial infarction (AMI). A retrospective cohort analysis using administrative databases kept by eight local health units was performed. DAPT exposure (defined as ≥ 2 prescriptions), adherence, and the occurrence of major adverse events (MACE) were analyzed during a 36-month follow-up. The analysis included 11,101 patients who were discharged alive with a primary diagnosis of AMI. Of these, 5,919 patients (53.31%) were discharged on DAPT without a diagnosis of cancer or anemia, without transient DAPT discontinuation, and represented the study population. DAPT discontinuation occurred in 2,200 patients (37.2%) and in 1,995 (33.7%) after the first 6 and 12 months, respectively, whereas 423 patients (7.1%) were still on DAPT after 36 months. Patients who maintained DAPT up to 12 months had a significantly lower overall mortality, compared with patients who discontinued DAPT after 6 months. Exposure to DAPT at 3 years was associated with reduced all-cause mortality (hazard ratio [HR]: 0.067, 95% confidence interval [CI]: 0.027–0.162, *p* < 0.001) and reduced recurrent AMI (HR: 0.02, 95% CI: 0.003–0.173, *p* < 0.001). In conclusion, this study shows that prolonged DAPT over 12 months is maintained in a relevant number of patients after AMI. However, adherence to antiplatelet therapy in first 12 months after AMI is still unsatisfactory and efforts to enhance patients' compliance are warranted. Exposure to prolonged DAPT at 3 years seems to be associated with a significant reduction in all-cause mortality and AMI.

## Introduction

Antiplatelet therapy represents the cornerstone therapy in patients with acute coronary syndrome (ACS). Prolonged dual-antiplatelet therapy (DAPT) with aspirin and a P2Y _12_ inhibitor is recommended up to 1 year after ACS both in patients submitted to revascularization and in patients medically managed. 1 2 3

Notably, only in the late years a mounting evidence for secondary prevention by intensified antiplatelet therapy has emerged after 1 year from ACS. It has been recently demonstrated that, in patients presenting with ACS, the ischemic risk can remain substantially elevated beyond the first 12 months, in spite of a successful revascularization. The DAPT 4 and the PEGASUS-TIMI 54 5 trials demonstrated that prolonged DAPT beyond 12 months can significantly reduce the incidence of major adverse cardiac events (MACE). In this setting, intensified antiplatelet therapy on top of aspirin has been shown to be an effective therapeutic strategy to prevent recurrent ischemic events. In selected patients at high thrombotic risk, prolonged DAPT beyond 12 months might be considered. 1 2 3

The need for prolonged DAPT might raise compliance issues, as long-term adherence to prescribed therapy after ACS is still challenging in real-world patients. 6

Previous reports have shown that more than 10% of patients prematurely discontinue antiplatelet therapy within 30 days after stent implantation. Clinical trials reported a rate of premature discontinuation as high as 30% after 12 months. 7 Premature antiplatelet therapy discontinuation raises safety concerns, as it is associated with a higher incidence of MACE. 6 8 Nonadherent patients are at a substantially higher risk of death. 9 Patients with ACS who discontinue all of their medications are more than three times as likely to die as those who remain adherent. Of note, the rate and clinical consequences of antiplatelet discontinuation beyond 12 months after ACS in real-world patients are still lacking.

The aim of our study is to assess long-term exposure and adherence to DAPT in patients with ACS in an Italian population. The occurrence of MACE in relation to maintenance of single- or dual-antiplatelet therapy has been investigated, as well.

## Methods

### Data Sources

The PIPER (Platelet-aggregation Inhibition: Persistence with treatment and cardiovascular Events in Real-world Study) is a retrospective, observational study, based on administrative databases of eight Italian Local Health Units (LHUs), in Lombardy, Tuscany, Puglia, and Campania, which includes approximately 5.6 million health-assisted individuals.

In particular, the following databases were used as data sources: the Health-Assisted Subjects' Database, containing patients' demographic data; Outpatients and Inpatients pharmaceutical drugs, providing information for each medication prescription, such as ATC (Anatomical-Therapeutic-Chemical) code of the drug purchased, number of packages, number of units per package, the dosages, unit cost per package, and prescription date; and Hospital Discharge Database, which includes all hospitalization data with discharge diagnosis codes classified according to the International Classification of Diseases, Ninth Revision, Clinical Modification (ICD-9-CM). The patient code in each database permitted electronic linkage with all the other databases. No identifiers related to patients were provided to the researchers. Informed consent was not required for using encrypted retrospective information. This study was notified to the local ethics committee in each participating LHU according to the Italian law regarding the conduct of observational analysis, and the LHU Ethics Committees approved the study. 10

### Study Populations

This was a retrospective cohort study that included all consecutive beneficiaries of each LHUs hospitalized and discharged alive with a primary diagnosis of acute myocardial infarction (AMI; ICD-9-CM: 410.xx) between January 1, 2010, and December 31, 2011 (enrollment period). The date of AMI discharge was identified as the index date, which represented the enrollment day of each individual patient, who was then followed up for 3 years after the index date (follow-up period).

Data on baseline characteristics, including demographics, risk factors, and medical history, were collected. Specifically, the treatments of interest were antihypertensive drugs (ATC codes: C02, C03, C08), oral hypoglycemic drugs and/or insulins (ATC code: A10), cardiac therapy (ATC code: C01), statins (ATC code: C10AA), beta-blocking agents (ATC code: C07), angiotensin-converting enzyme (ACE) inhibitors/angiotensin II receptor antagonists (AAIIs; ATC code: C09). Previous cardiovascular hospitalizations were identified by ICD-9-CM codes: 410–414; history of percutaneous coronary intervention (PCI) (procedure codes: 00.66, 36.0x or absence of 36.04) and AMI of other than anterior wall (ICD-9-CM codes: 410.0 or 410.1) were also evaluated.

Patients were classified according to exposure to antiplatelet therapy during the follow-up period. A patient was defined as treated with antiplatelet therapy (ATC code: B01AC), if at least two prescriptions of these drugs during the follow-up period were found. In particular, all enrolled patients were stratified into four main categories: (1) monotherapy with acetylsalicylic acid (ASA); (2) DAPT (ASA + P2Y _12_ inhibitors); (3) other drugs (only P2Y _12_ inhibitors); and (iv) no antiplatelet therapy.

Patients with concomitant diagnosis at discharge of anemia or cancer were excluded, as these comorbidities might influence prescription and/or duration of DAPT.

Hospitalizations for AMI and all-cause mortality occurring during the 36-month follow-up period from discharge were considered. Multivariable analyses were conducted to check for possible confounders using a proportional hazards Cox regression model: demographic characteristics; other guidelines-recommended post-AMI therapies, including beta-blockers, ACE inhibitors/AAIIs, statin, and antiplatelet therapy; previous hospitalizations for cardiovascular disorders; and diabetes.

A patient was defined as antidiabetic, antihypertensive, or cardiac therapy if at least two prescriptions of antidiabetic agents, of antihypertensives, of cardiac drugs, respectively, were found at follow-up. A patient was defined as treated with statins, beta-blocking agents, or ACE inhibitors/AAIIs if at least two prescriptions of these drugs were detected during the observation period. Patients who were transferred to another LHU during the observational period were excluded from analysis.

A Pegasus score was calculated for each patient by the sum of the characteristics which constituted the inclusion criteria of the Pegasus trial. 5

### Statistical Methods

Continuous variables are given as means with standard deviations (mean ± SD); categorical variables are shown as percentages and absolute numbers.

Cox regression analysis has been used for the risk assessment. The impact of different variables on cardiovascular mortality and recurrent AMI was analyzed using the hazard ratios (HR) with 95% confidence interval (CI); a *p* -value less than 0.05 was considered statistically significant. Analyses were performed with Stata software version 12.1 (Stata Corp LP, College Station, Texas, United States).

## Results

**Fig. 1:**
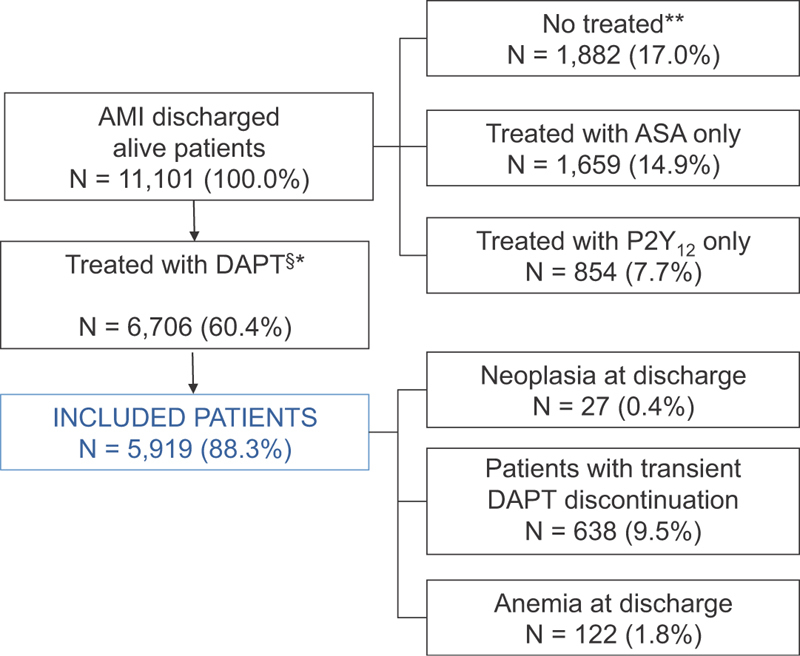
Flow chart for patient selection and inclusion and exclusion criteria for study. DAPT, dual-antiplatelet therapy; AMI, acute myocardial infarction; ASA, acetylsalicylic acid. *Notes* : ^§^ DAPT = ASA + P2Y _12_ inhibitors (clopidogrel, prasugrel, ticagrelor, ticlopidine); *At least one prescription of ASA and at least one prescription of P2Y _12_ in the month following hospital discharge; **Any prescription of ASA or P2Y _12_ in the 3 months following hospital discharge.

A total of 11,101 were discharged alive with a primary diagnosis of AMI. Of these, 5,919 patients (53.3%) were discharged on DAPT, without a diagnosis of cancer or anemia at discharge, without transient DAPT discontinuation, and represented our study population ( Fig. 1 ).

The demographic and baseline clinical characteristics of included patients are shown in Table 1 . Among the enrolled patients, 55.6% presented hypertension at the baseline, followed by 43.9% with heart disease, 26.0% with diabetes, and 10.0% with previous hospitalization for cardiovascular disorders. Statins, ACE inhibitors/AAIIs, and beta-blocking agents were used in 79.4, 74.6, and 71.5%, respectively. PCI at the index hospitalization was performed in 52.9% of patients. Table 2 describes the type of DAPT.

**Table 1:** Demographic and clinical characteristics of included patients

*N* , %	11,101
	100.0%
Age (y)	65.6 ± 17.6
Male	7,000
	63.1%
Previous cardiovascular hospitalizations	1,114
	10.0%
Diabetes	2,885
	26.0%
Hypertension	6,167
	55.6%
Heart disease	4,878
	43.9%
ACE inhibitors/AAIIs	8,285
	74.6%
Statins	8,819
	79.4%
Beta-blocking agents	7,942
	71.5%
Index MI: front/front-lateral	2,889
	26.0%
Index MI with PCI	5,872
	52.9%
Index MI with coronary angiography	5,821
	52.4%
CKD	232
	2.1%

Abbreviations: AAII, angiotensin II receptor antagonist; ACE, angiotensin-converting enzyme; CKD, chronic kidney disease; MI, myocardial infarction; PCI, percutaneous coronary intervention.

**Table 2:** Type of antiplatelet treatment

Type of treatment			*N*	%	%
None			1,882	17.0	–
Treatment			9,219	83.0	100.0
	Monotherapy	ASA	1,659	14.9	18.0
		P2Y12	854	7.7	9.3
	DAPT	ASA + Clopidogrel	6,313	56.9	68.4
		ASA + Prasugrel	266	2.4	2.9
		ASA + Ticagrelor	10	0.1	0.1
		ASA + Ticlopidine	117	1.1	1.3
Total				100.0	–

Abbreviations: ASA, acetylsalicylic acid; DAPT, dual-antiplatelet therapy (ASA + P2Y _12_ ).

Out of the 5,919 patients, 2,200 (37.2%) discontinued DAPT after the first semester and 1,995 (33.7%) discontinued DAPT after the second semester ( Supplementary Fig. S1 ). Of note, 423 patients (7.1%) were still on DAPT after 36 months.

Baseline clinical and demographic characteristics of patients in relation to exposure and type of antiplatelet therapy are summarized in Table 3 . Compared with patients who were still on DAPT at 6 months, patients who were on DAPT at 12 months were younger (mean age: 62.5 ± 16.0 vs. 65.5 ± 17.0, *p* < 0.001), less frequently required blood transfusion (3.9 vs. 6.9%, *p* < 0.001), and had less frequently chronic kidney disease (1.1 vs. 2.2%, *p* < 0.01). Patients who were still on DAPT at 3 years had more frequently a previous cardiovascular hospitalization (8.8 vs. 14.4%, *p* < 0.001) and were more frequently diabetic (38.5 vs. 25.5%, *p* < 0.001), compared with patients who discontinued after 12 months.

**Table 3:** Demographic and clinical characteristics of patients by semester of DAPT interruption

	Patients who discontinued DAPT after semester 1	Patients who discontinued DAPT after semester 2	Patients who discontinued DAPT after semester 3	Patients who discontinued DAPT after semester 4	Patients who discontinued DAPT after semester 5	Patients still in DAPT at semester 6	*p* -Value
*N* , %	2,200	1,995	807	324	170	423	
	37.2%	33.7%	13.6%	5.5%	2.9%	7.1%	
Age (y)	65.5 ± 17.0	62.5 ± 16.0	61.4 ± 15.9	62.0 ± 17.6	61.8 ± 16.9	61.7 ± 17.9	0.001
Male	1,434	1,424	558	211	119	275	
	65.2%	71.4%	69.1%	65.1%	70.0%	65.0%	0.01
Previous cardiovascular hospitalizations	193	161	62	33	22	61	0.001
	8.8%	8.1%	7.7%	10.2%	12.9%	14.4%	
Diabetes	560	532	250	92	62	163	0.001
	25.5%	26.7%	31.0%	28.4%	36.5%	38.5%	
Hypertension	1,322	984	478	219	112	291	0.001
	60.1%	49.3%	59.2%	67.6%	65.9%	68.8%	
Heart disease	1,045	774	372	189	103	251	0.001
	47.5%	38.8%	46.1%	58.3%	60.6%	59.3%	
ACE inhibitors/AAIIs	1,806	1,718	726	283	156	382	0.001
	82.1%	86.1%	90.0%	87.3%	91.8%	90.3%	
Statins	1,946	1,938	793	320	164	413	0.001
	88.5%	97.1%	98.3%	98.8%	96.5%	97.6%	
Beta-blocking agents	1,736	1,710	693	282	154	365	0.001
	78.9%	85.7%	85.9%	87.0%	90.6%	86.3%	
Index MI: front/front-lateral	583	541	206	82	61	122	NS
	26.5%	27.1%	25.5%	25.3%	35.9%	28.8%	
Index MI with PCI	1,368	1,425	534	195	107	266	0.001
	62.2%	71.4%	66.2%	60.2%	62.9%	62.9%	
Index MI with coronary angiography	1,260	1,294	468	175	89	234	0.001
	57.3%	64.9%	58.0%	54.0%	52.4%	55.3%	
CKD	48	21	9	5	10	8	0.001
	2.2%	1.1%	1.1%	1.5%	5.9%	1.9%	
	0.3%	0.4%	–	–	0.0%	1.4%	

Abbreviations: AAII, angiotensin II receptor antagonist; ACE, angiotensin-converting enzyme; CKD, chronic kidney disease; DAPT, dual-antiplatelet therapy; MI, myocardial infarction; NI, not issuable; PCI, percutaneous coronary intervention.

**Fig. 2:**
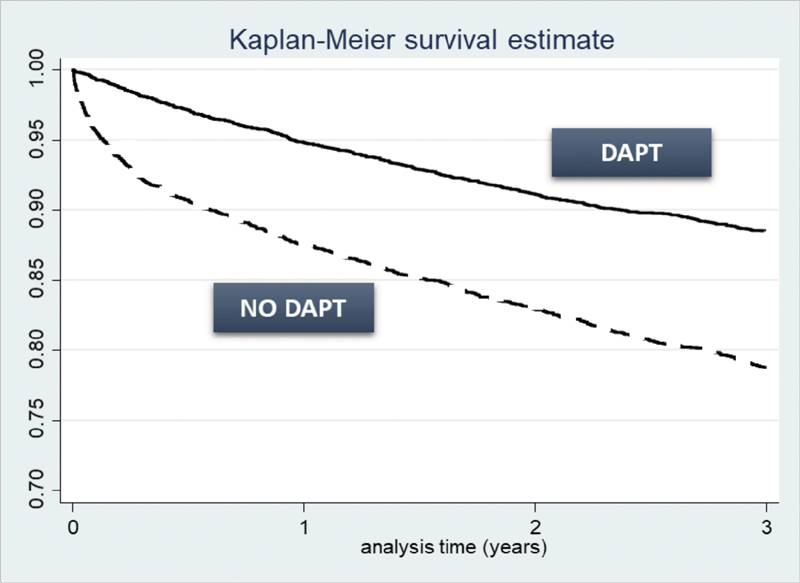
Mortality at 36 months of follow-up (Kaplan–Meier). NO DAPT: N = 3,540, no. of events = 749 (21.2%); survivals after 3 years = 2,791 (78.8%); DAPT: N = 5,027, no. of events = 580 (11.6%); survivals after 3 years = 4,447 (88.4%). LHUs of Frosinone and Grosseto were excluded because data on deaths were not available.

After controlling for possible confounders, patients who maintained DAPT up to 12 months had with a significantly lower all-cause mortality, compared with patients who discontinued DAPT after 6 months (HR: 0.531, 95% CI: 0.434–0.650, *p* < 0.001). Moreover, exposure to DAPT at 3 years (compared with no exposure to DAPT—no treated patients or patients in monotherapy with ASA/P2Y12) was associated with reduced all-cause mortality (HR: 0.067, 95% CI: 0.027–0.162, *p* < 0.001; Fig. 2 ) and recurrent AMI (HR: 0.02, 95% CI: 0.003–0.173, *p* < 0.001). PCI at the index hospitalization for AMI was found to be associated with reduced all-cause mortality (HR: 0.678, 95% CI: 0.557–0.825, *p* < 0.001), but was not associated with reduced AMI ( Table 4 ).

**Table 4:** All-cause mortality at 3 years in relation to DAPT maintenance

	HR	95% confidential interval		*p* -Value
Age (y)	1.076	1.064	1.087	<0.001
Gender (ref. F)	1.576	1.316	1.886	<0.001
Previous cardiovascular hospitalizations (ref. no)	1.257	0.965	1.636	0.090
Diabetes (ref. no)	0.972	0.689	1.371	N.S.
Hypertension (ref. no)	0.820	0.579	1.160	N.S.
Heart disease (ref. no)	0.735	0.534	1.013	N.S.
ACE inhibitors/AAIIs (ref. no)	0.235	0.168	0.328	<0.001
Statins (ref. no)	0.574	0.458	0.720	<0.001
Beta-blocking agents (ref. no)	0.417	0.301	0.578	<0.001
Index MI: front/front-lateral (ref. no)	1.058	0.872	1.282	N.S.
Index MI with PCI (ref. no)	0.678	0.557	0.825	<0.001
Index MI with coronary angiography (ref. no)	0.950	0.783	1.153	N.S.
Neoplasia hosp. during obs. period (ref. no)	2.992	2.421	3.697	<0.001
Blood transfusion during obs. period (ref. no)	1.376	0.949	1.997	N.S.
DAPT maintenance				
Patients who discontinued DAPT after semester 1	1.000			
Patients who discontinued DAPT after semester 2	0.531	0.434	0.650	**<0.001**
Patients who discontinued DAPT after semester 3	0.455	0.330	0.628	**<0.001**
Patients who discontinued DAPT after semester 4	0.484	0.323	0.725	**0.001**
Patients who discontinued DAPT after semester 5	0.195	0.096	0.395	**<0.001**
Patients still in DAPT at semester 6	0.067	0.027	0.162	**<0.001**

Abbreviations: AAII, angiotensin II receptor antagonist; ACE, angiotensin-converting enzyme; DAPT, dual-antiplatelet therapy; HR, hazard ratio; MI, myocardial infarction; N.S., not significant; PCI, percutaneous coronary intervention.

Notes: *N* = 5,027, no. of events = 580.

## Discussion

The main findings of this study are the following: adherence to DAPT within the first 12 months after AMI is still suboptimal; notably, a significant proportion of patients in Italy maintain DAPT up to 36 months after AMI; cessation of DAPT within the first 12 months is associated with increased overall mortality; prolonged DAPT beyond 12 months seems to be associated with a significant reduction in all-cause mortality and AMI.

The benefit of DAPT after an ACS was well demonstrated in the CURE, 11 COMMIT/CCS-28, 12 and CLARITY-TIMI 28 13 trials. The association of aspirin and clopidogrel reduced the 1-year incidence of cardiovascular events by approximately 20% compared with aspirin alone. More potent and consistent P2Y _12_ receptor inhibition with either prasugrel or ticagrelor was superior to clopidogrel in the subsequent TRITON-TIMI 38 14 and PLATO 15 trials. Current guidelines recommend 12 months of DAPT after ACS. 1 2 3 These recommendations are made on the basis of early studies 16 17 which demonstrated a sustained increased risk of thrombotic complications, including stent thrombosis and spontaneous cardiovascular events, beyond 6 months. Notably, the greatest absolute reductions in cardiovascular events with DAPT are seen in the first 3 months and since these studies, advances in drug-eluting stent technology have led to a substantially reduced incidence of late (>30 days) and very late (>1 year) stent thrombosis. 10 18

Although it has been fully elucidated that premature discontinuation of antiplatelet therapy is associated with a poor prognosis, patients' compliance remains low. 6 7 Several reasons for noncompliance have been reported, such as excessive concerns about hemorrhagic complications or competing guidelines, complex treatment regimens, or lack of outcome expectancy. 19 As anemia can be considered a marker for high bleeding risk, thus contraindicating prolonged DAPT, patients with anemia at discharge were excluded from the present analysis. Nevertheless, in this study, one out of three patients discontinued DAPT after 6 months. In routine clinical practice, poor medication adherence is one of the main factors that reduce the effectiveness of chronic drug therapies. 20 In accordance with data observed in other studies, 6 21 22 in this study a considerable proportion of patients report low adherence to prescribed medications, particularly for non-monotherapy strategies.

Although many studies have evaluated the incidence or effect of antiplatelet therapy cessation on subsequent cardiovascular risk, most included selected cohorts of patients and were limited by lack of standardized definitions for therapy discontinuation that did not integrate the underlying context in which treatment was discontinued. 23 24 The results presented here suggest that there are still numerous barriers to adherence to practice guidelines; therefore, encouragement to continue long-term therapy with lifestyle modifications and pharmacological treatments is needed to improve outcomes in patients at risk of cardiovascular events. 25

On the other hand, this study demonstrated that the number of patients who maintain DAPT up to 3 years after AMI is not trivial. It should be highlighted that the enrollment period was from January 2010 to December 2011, previous to the recent publication of data on prolonged DAPT beyond 12 months. This finding might be considered in the light of the attempt of clinicians to provide a further protection for ischemic events in patients considered high thrombotic risk, even without evidence of clear benefit. The DAPT trial was designed on the basis of this unmet need. In the DAPT trial, 4 the largest and only double-blinded study, extended DAPT (30 vs. 12 months) reduced the risk of major adverse cardiovascular and cerebrovascular events (4.3 vs. 5.9%), myocardial infarction (MI; 2.1 vs. 4.1%), and stent thrombosis (0.4 vs. 1.4%) but at a cost of increased moderate or severe bleeding (2.5 vs. 1.6%) and a borderline rise in all-cause mortality (2.0 vs. 1.0%; *p* = 0.05). The PEGASUS-TIMI 54 trial 5 compared aspirin monotherapy to a combination of aspirin and ticagrelor in patients with a previous MI and at least one additional high-risk factor. At a mean of 33 months, ticagrelor (60 mg) reduced the incidence of cardiovascular death, MI, or stroke (7.77 vs. 9.04%) at the expense of increased thrombolysis in myocardial infarction (TIMI) major bleeding (2.30 vs. 1.06%) and a neutral effect on overall mortality. On the basis of these trials, combination antiplatelet therapy would appear to confer only a small ischemic benefit at the cost of a significant bleeding risk. European 26 and North American 27 guidelines therefore do not recommend DAPT in patients with stable atherothrombotic disease but acknowledge that with careful consideration, combined antiplatelet therapy may be beneficial in some high-risk patients.

In this study, prolonged DAPT up to 3 years after AMI was associated with a significant reduction in overall mortality and AMI. Due to the retrospective nature of the study, this conclusion should be interpreted with caution and only as hypothesis generating. It should be highlighted that prolonged DAPT might identify patient at lower hemorrhagic risk, without noncardiac comorbidities which would have contraindicated long-term DAPT. To reduce a selection bias, patients with anemia of cancer at discharge were excluded, as well as patients who were not prescribed DAPT at discharge. An unexpected finding in the DAPT trial 4 was a borderline-significant increase in overall mortality rate (0.5% absolute increase) with 30 months of DAPT versus 12 months of DAPT in DES-treated patients, which was due to significantly increased deaths from non-cardiovascular causes (most commonly cancer), with no increase in cardiovascular deaths, and no significant increase in fatal bleeding.

It should be highlighted that the prescription of DAPT at discharge was relatively low. This might be partly explained by the enrollment period, which was from January 2010 to December 2011, when P2Y _12_ inhibitors in medically managed patients were still underused. Moreover, the analysis included not only type I but also type II MI patients, for whom the benefit of DAPT may be controversial. The low use of beta-blockers and ACE inhibitors was evident, as well. The low use of ACE inhibitors might be due in some cases to preserved ejection fraction.

The results of this study should be interpreted in light of some strengths and limitations. The strength of the presented data includes its basis on the large size unselected population; our patients may represent a more representative sample of “real world” than those included in many of the randomized controlled trials that have previously evaluated antiplatelet use for mortality reduction. Limitations include, first, the absence of relevant clinical information in the data setting. It is plausible that patients' risk profile might have conditioned DAPT duration. However, due to the nature of the study, which consists of a retrospective analysis from administrative databases, important clinical data are missing and confounding biases might have influenced the results. To overcome this limitation, we excluded patients with concomitant diagnosis at discharge of anemia or cancer, as these comorbidities might influence prescription and/or duration of DAPT. It cannot be ruled out if the reduced all-cause mortality associated with prolonged exposure to DAPT is related to a lower risk profile of patients (in terms of comorbidities), which allowed prolonged DAPT, or the protective effect of DAPT. Notably, prolonged DAPT was associated with a reduced rate of recurrent AMI, for which the protective effect of DAPT might be more evident. Second, adherence was estimated using pharmacy data on filled prescriptions, but no information on actual medication consumption was available. Third, the reasons for nonadherence were not retrievable from the dataset.

Despite these limitations, this study indicates prolonged DAPT beyond 12 months is maintained in a relevant number of patients after AMI. Notably, long-term exposure and adherence with antiplatelet therapy in patients with AMI still appear to be unsatisfactory in an unselected Italian population. Further efforts to enhance patients' compliance to medical therapy are warranted.

## References

[1] Valgimigli M Bueno H Byrne R A 2017 ESC focused update on dual antiplatelet therapy in coronary artery disease developed in collaboration with EACTS: The Task Force for dual antiplatelet therapy in coronary artery disease of the European Society of Cardiology (ESC) and of the European Association for Cardio-Thoracic Surgery (EACTS) Eur Heart J 2018 39 03 213 260 28886622 10.1093/eurheartj/ehx419

[2] Ibanez B James S Agewall S 2017 ESC Guidelines for the management of acute myocardial infarction in patients presenting with ST-segment elevation: the Task Force for the management of acute myocardial infarction in patients presenting with ST-segment elevation of the European Society of Cardiology (ESC) Eur Heart J 2018 39 02 119 177 28886621 10.1093/eurheartj/ehx393

[3] Hamm C W Bassand J-P Agewall S ESC Guidelines for the management of acute coronary syndromes in patients presenting without persistent ST-segment elevation: The Task Force for the management of acute coronary syndromes (ACS) in patients presenting without persistent ST-segment elevation of the European Society of Cardiology (ESC) Eur Heart J 2011 32 23 2999 3054 21873419 10.1093/eurheartj/ehr236

[4] Kereiakes D J Yeh R W Massaro J M Antiplatelet therapy duration following bare metal or drug-eluting coronary stents: the dual antiplatelet therapy randomized clinical trial JAMA 2015 313 11 1113 1121 25781440 10.1001/jama.2015.1671 PMC4481320

[5] Bonaca M P Bhatt D L Cohen M Long-term use of ticagrelor in patients with prior myocardial infarction N Engl J Med 2015 372 19 1791 1800 25773268 10.1056/NEJMoa1500857

[6] Mathews R Peterson E D Honeycutt E Early medication nonadherence after acute myocardial infarction: insights into actionable opportunities from the TReatment with ADP receptor iNhibitorS: Longitudinal Assessment of Treatment Patterns and Events after Acute Coronary Syndrome (TRANSLATE-ACS) study Circ Cardiovasc Qual Outcomes 2015 8 04 347 356 26038524 10.1161/CIRCOUTCOMES.114.001223 PMC4512913

[7] Bonaca M P Bhatt D L Oude Ophuis T Long-term tolerability of ticagrelor for the secondary prevention of major adverse cardiovascular events: a secondary analysis of the PEGASUS-TIMI 54 trial JAMA Cardiol 2016 1 04 425 432 27438319 10.1001/jamacardio.2016.1017

[8] Rossini R Capodanno D Lettieri C Prevalence, predictors, and long-term prognosis of premature discontinuation of oral antiplatelet therapy after drug eluting stent implantation Am J Cardiol 2011 107 02 186 194 21211596 10.1016/j.amjcard.2010.08.067

[9] Rasmussen J N Chong A Alter D A Relationship between adherence to evidence-based pharmacotherapy and long-term mortality after acute myocardial infarction JAMA 2007 297 02 177 186 17213401 10.1001/jama.297.2.177

[10] Wilson S J Newby D E Dawson D Irving J Berry C Duration of dual antiplatelet therapy in acute coronary syndrome Heart 2017 103 08 573 580 28249994 10.1136/heartjnl-2016-309871 PMC5529971

[11] Yusuf S Zhao F Mehta S R Chrolavicius S Tognoni G Fox K K; Clopidogrel in Unstable Angina to Prevent Recurrent Events Trial Investigators.Effects of clopidogrel in addition to aspirin in patients with acute coronary syndromes without ST-segment elevation N Engl J Med 2001 345 07 494 502 11519503 10.1056/NEJMoa010746

[12] Chen Z M Jiang L X Chen Y P Addition of clopidogrel to aspirin in 45,852 patients with acute myocardial infarction: randomised placebo-controlled trial Lancet 2005 366(9497):1607 1621 16271642 10.1016/S0140-6736(05)67660-X

[13] Sabatine M S Cannon C P Gibson C M Addition of clopidogrel to aspirin and fibrinolytic therapy for myocardial infarction with ST-segment elevation N Engl J Med 2005 352 12 1179 1189 15758000 10.1056/NEJMoa050522

[14] Wiviott S D Braunwald E McCabe C H Prasugrel versus clopidogrel in patients with acute coronary syndromes N Engl J Med 2007 357 20 2001 2015 17982182 10.1056/NEJMoa0706482

[15] Wallentin L Becker R C Budaj A Ticagrelor versus clopidogrel in patients with acute coronary syndromes N Engl J Med 2009 361 11 1045 1057 19717846 10.1056/NEJMoa0904327

[16] Pfisterer M Brunner-La Rocca H P Buser P T Late clinical events after clopidogrel discontinuation may limit the benefit of drug-eluting stents: an observational study of drug-eluting versus bare-metal stents J Am Coll Cardiol 2006 48 12 2584 2591 17174201 10.1016/j.jacc.2006.10.026

[17] Eisenstein E L Anstrom K J Kong D F Clopidogrel use and long-term clinical outcomes after drug-eluting stent implantation JAMA 2007 297 02 159 168 17148711 10.1001/jama.297.2.joc60179

[18] Palmerini T Biondi-Zoccai G Della Riva D Clinical outcomes with drug-eluting and bare-metal stents in patients with ST-segment elevation myocardial infarction: evidence from a comprehensive network meta-analysis J Am Coll Cardiol 2013 62 06 496 504 23747778 10.1016/j.jacc.2013.05.022

[19] Stafford R S Radley D C The underutilization of cardiac medications of proven benefit, 1990 to 2002 J Am Coll Cardiol 2003 41 01 56 61 12570945 10.1016/s0735-1097(02)02670-0

[20] Marcum Z A Sevick M A Handler S M Medication nonadherence: a diagnosable and treatable medical condition JAMA 2013 309 20 2105 2106 23695479 10.1001/jama.2013.4638 PMC3976600

[21] Faxon D Brown M Antiplatelet therapy for postdischarge medical management of acute coronary syndrome Am J Med 2008 121 03 171 178 18328296 10.1016/j.amjmed.2007.09.026

[22] Lenzi J Rucci P Castaldini I Does age modify the relationship between adherence to secondary prevention medications and mortality after acute myocardial infarction? A nested case-control study Eur J Clin Pharmacol 2015 71 02 243 250 25529226 10.1007/s00228-014-1793-8

[23] Brar S S Kim J Brar S K Long-term outcomes by clopidogrel duration and stent type in a diabetic population with de novo coronary artery lesions J Am Coll Cardiol 2008 51 23 2220 2227 18534267 10.1016/j.jacc.2008.01.063

[24] Daemen J Wenaweser P Tsuchida K Early and late coronary stent thrombosis of sirolimus-eluting and paclitaxel-eluting stents in routine clinical practice: data from a large two-institutional cohort study Lancet 2007 369(9562):667 678 17321312 10.1016/S0140-6736(07)60314-6

[25] Brown M T Bussell J K Medication adherence: WHO cares? Mayo Clin Proc 2011 86 04 304 314 21389250 10.4065/mcp.2010.0575 PMC3068890

[26] Montalescot G Sechtem U Achenbach S 2013 ESC guidelines on the management of stable coronary artery disease: the Task Force on the management of stable coronary artery disease of the European Society of Cardiology Eur Heart J 2013 34 38 2949 3003 23996286 10.1093/eurheartj/eht296

[27] Levine G N Bates E R Bittl J A 2016 ACC/AHA Guideline Focused Update on Duration of Dual Antiplatelet Therapy in Patients With Coronary Artery Disease: A Report of the American College of Cardiology/American Heart Association Task Force on Clinical Practice Guidelines: An Update of the 2011 ACCF/AHA/SCAI Guideline for Percutaneous Coronary Intervention, 2011 ACCF/AHA Guideline for Coronary Artery Bypass Graft Surgery, 2012 ACC/AHA/ACP/AATS/PCNA/SCAI/STS Guideline for the Diagnosis and Management of Patients With Stable Ischemic Heart Disease, 2013 ACCF/AHA Guideline for the Management of ST-Elevation Myocardial Infarction, 2014 AHA/ACC Guideline for the Management of Patients With Non-ST-Elevation Acute Coronary Syndromes, and 2014 ACC/AHA Guideline on Perioperative Cardiovascular Evaluation and Management of Patients Undergoing Noncardiac Surgery Circulation 2016 134 10 e123 e155 27026020 10.1161/CIR.0000000000000404

