# Supplementary material for: Long-Term Use of Antiplatelet Therapy in Real-World Patients with Acute Myocardial Infarction: Insights from the PIPER Study
Source: TH Open. 2018 Dec 21;2(4):e437–44. doi: 10.1055/s-0038-1676529 (PMC6524921; doi:10.1055/s-0038-1676529)
Supplement: Supplementary file 1 — Supplementary Material [file 10-1055-s-0038-1676529-s180013.pdf]

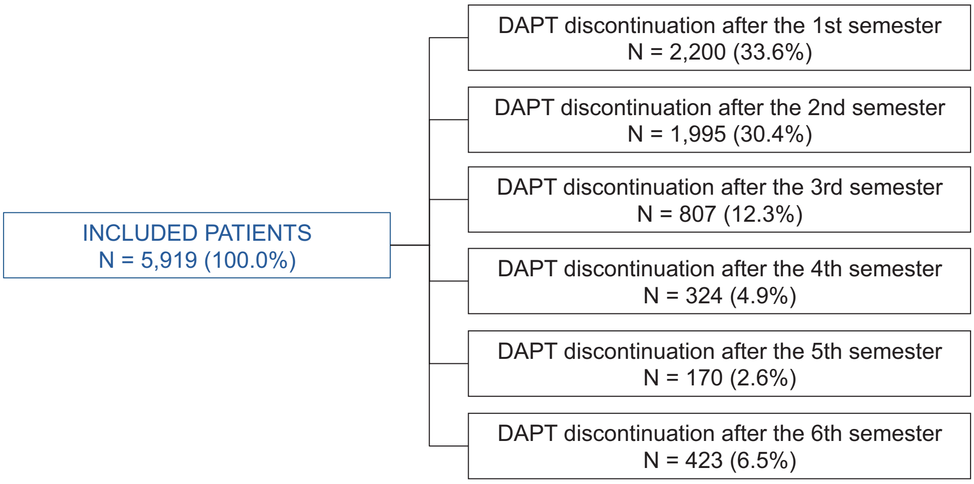

Supplementary Fig. S1 Time of DAPT discontinuation among included patients. DAPT, dual-antiplatelet therapy (at least one prescription of ASA and at least one prescription of $P2Y_{12}$ in the month following hospital discharge).
